# The panacea toolbox of a PhD biomedical student

**DOI:** 10.12669/pjms.306.6378

**Published:** 2014

**Authors:** Younis Skaik

**Keywords:** PhD, Skills

## Abstract

Doing a PhD (doctor of philosophy) for the sake of contribution to knowledge should give the student an immense enthusiasm through the PhD period. It is the time in one’s life that one spends to “hit the nail on the head” in a specific area and topic of interest. A PhD consists mostly of hard work and tenacity; however, luck and genius might also play a little role. You can pass all PhD phases without having both luck and genius. The PhD student should have pre-PhD and PhD toolboxes, which are “sine quibus non” for getting successfully a PhD degree. In this manuscript, the toolboxes of the PhD student are discussed.

## INTRODUCTION

The PhD (doctor of philosophy) pathway is full of highs and lows, and should terminate with a valuable contribution to knowledge. During this pathway, one should demonstrate one’s ability to conduct independently scholarly research, and the ability to solve research problems to reach the “Cinderella moment”. To keep the research steps in the correct orbit, toolboxes are needed to navigate the PhD journey. Most biomedical students decide to go through the PhD journey either to improve their academic careers, seek the intellectual challenge or to satisfy their eager about a certain topic. The reason that spurred one to pursue the PhD will determine the shape and fate of your PhD thesis and contribution to knowledge. To better understand the PhD process and the pre-and PhD tools which are needed during the journey, the panacea toolboxes are discussed in this manuscript.

***Pre-PhD toolbox:*** Before one takes a step into the PhD world, one should make sure first it is really what one want. Second, you should have list of skills to make the way unclouded through the PhD journey. Communication skills such as academic writing, reading papers and how to evaluate a paper are very important skills that would give the biomedical student the ability to touch the strengths and weaknesses of each paper related to his topic, and how to find the gaps in the previous research and how to fill the gaps in his future research. Independent work and creativity are also needed. Table-I shows the top 10 skills that the biomedical student should have to make the way easier, as one have several tasks over a tight time through the PhD journey.

**Table-I:** Top 10 skills of a pre-PhD student.

**No.**	**Skill**	**Description**
**1**	**Creativity**	Seven key questions (who, what, why, when, where, how and how much) cover the basics and help you to understand and figure out a situation.
**2**	**Research methods**	Qualitative, quantitative and cohort studies. Sampling techniques. All of those and many others should be familiar to the biomedical students.
**3**	**Search medical databases**	Skills of searching effectively the databases are needed to achieve highly sensitive and specific results.^1^
**4**	**Reading a paper**	How to read and interpret a paper in your field is highly-demanded.^2^
**5**	**Writing a paper**	Improve your writing skills as much as you can.^3^
**6**	**Computer skills**	Microsoft’s word, PowerPoint and Excel skills are all desired during your PhD journey.
**7**	**Presenting results**	How to present your results to academics and non-academics is an art, which needs training and previous knowledge.^4^
**8**	**Independent work**	One should be used to organize the daily research without having to be alerted.
**9**	**Documenting and reporting**	Learn how to keep everything documented and reported would help you so much during the PhD degree.
**10**	**Statistical knowledge**	It is always preferred to have some knowledge about statistics and the corresponding softwares.

***PhD toolbox: ***"If we knew what it was we were doing, it would not be called research, would it?" It is a transition time that you spend during the PhD period to go day by day to higher orbitals armed with more and more skills, which will enable you to “hit the nail on the head” in a specific area and topic of interest, and should terminate with a valuable contribution to knowledge. First of all and before you decide to go through the PhD journey, one should choose a supervisor who is interested and has done some work in that particular area and who is productive as well. To improve one’s skills during the PhD period, start first with reading and evaluating the literature to enable you to identify the previous problems, how to analyze and solve them. The top 10 tips for improving the PhD skills and for passing successfully the journey are highlighted in Table-II.

**Table-II:** Top 10 tips of a PhD student.

**No.**	**Tip**	**Description**
**1**	**Time management**	Plan you days and weeks and try to stick to your plans.
**2**	**Reading and analyzing the literature**	This will help you a lot to define the problems and to solve the problems.
**3**	**Patience**	You should have a high patience threshold.
**4**	**Attending seminars and conferences**	To extend your knowledge and creativity. Be always active and feel free to ask questions.
**5**	**Practice writing**	To increase and improve your ability to write your first paper and the thesis.
**6**	**Talk about your project**	Talk about your projects and the problems you face to your colleagues and your supervisor. This will always open new vistas.
**7**	**Take courses**	Courses such as statistics, communication skills, English, or any course you feel that you need it to improve your career.
**8**	**Join international and professional societies**	This will keep you in the loop in your topic and field.
**9**	**Talk to the sales representatives**	You will always learn new diagnostic things from them.
**10**	**Take some days off and enjoy the formal holidays**	It will make your brain always fresh to re-think and evaluate the projects’ troubles.

## References

[1] Skaik Y (2013). Medical literature review: search or perish. Pak J Med Sci.

[2] Greenhalgh T (1997). How to read a paper Papers that report diagnostic or screening tests. BMJ.

[3] Katz MJ (2009). From Research to Manuscript: A Guide to Scientific Writing.

[4] Hites R (2014). How to give a scientific talk, present a poster, and write a research paper or proposal. Environ Sci Technol.

